# An enhanced performance analysis of load based resource sharing framework for MIMO systems in 5G communication systems

**DOI:** 10.1038/s41598-025-06441-8

**Published:** 2025-07-02

**Authors:** J. Logeshwaran, Shobhit K. Patel, Om Prakash Kumar, Fahad Ahmed Al-Zahrani

**Affiliations:** 1 Department of Computer Science, Christ University, Bengaluru, Karnataka 560029 India; 2 Department of Computer Engineering, Marwadi University, Rajkot, 360003 India; 3 Department of Electronics and Communication Engineering, Manipal Institute of Technology, Manipal Academy of Higher Education, Manipal, 576104 India; 4 Computer Engineering Department, Umm Al-Qura University, 24381 Mecca, Saudi Arabia

**Keywords:** Network, Resource, MIMO, 5G, Communication system, Load, Channel, Terminal access, SDG 9 (industry, innovation, and infrastructure), SDG 11 (sustainable cities and communities), SDG 12 (responsible consumption and production), Electrical and electronic engineering, Engineering, Mathematics and computing, Computer science

## Abstract

Resource sharing serves as a cost-effective and dynamically adjustable method for alleviating traffic congestion in wireless networks. Advancements in multi-input multi-output (MIMO) technologies for 5G communication systems have led to the exploration of resource sharing across various cells or sectors. This approach aims to optimise network performance, focussing on coverage, capacity, and quality of service. This document presents a new load-based resource-sharing framework designed for multi-cell MIMO systems. The proposed framework utilises channel-loading data from local base stations and dynamically allocates available resources among adjacent base stations. The proposed framework facilitates dynamic resource sharing, effectively addressing traffic overload in 5G networks. The proposed LBRS achieved a delta-P value of 90.91%, a prevalence threshold value of 89.84%, a critical success index value of 91.01%, and a Mathew’s correlation coefficient value of 91.27% at the terminal access. At the resource transmission, the system recorded a delta-P value of 92.10%, a prevalence threshold value of 92.18%, a critical success index value of 91.65%, and a Mathew’s correlation coefficient value of 88.31%. The simulation results indicate that the proposed framework effectively enhances dynamic resource sharing, resulting in a notable improvement in network performance.

## Introduction

MIMO represents a sophisticated technology implemented in contemporary 5G communication systems. This technology is utilised in the development of next-generation networks, enhancing performance by providing higher throughput and improved reliability, which results in more efficient utilisation of spectrum resources^1^. The system incorporates multiple antennae to facilitate communication across various paths, thereby enhancing its capability to manage multipath propagation effectively. MIMO systems utilise multiple antennas to generate several data streams over a single channel. It facilitates the concurrent transmission and reception of data. The implementation of 5G communication systems allows for the simultaneous transmission and reception of multiple data streams, thereby enhancing processing power and speed^2^. The primary advantage of MIMO technology lies in its capacity to enhance data transmission and reception within a single channel. This mechanism compensates for multipath propagation, which frequently results in reflective signals that may lead to interference and signal dropout, thereby enhancing the reliability and security of communication.

Additionally, it enhances spectral efficiency, allowing for the transmission and reception of multiple data streams within the same bandwidth^3^. MIMO technology contributes to the reduction of latency, defined as the delay encountered in the transmission of data between two points. Maintaining low latency is a critical requirement for 5G networks to guarantee high-quality service. MIMO technology can effectively reduce latency by enabling faster data processing through the utilisation of multiple antennas^4^. MIMO represents a critical technology integral to the functionality of contemporary 5G communication systems. MIMO enhances network performance and reliability by utilising multiple antennas to facilitate multiple data streams, thereby increasing spectral efficiency^5^. Furthermore, it contributes to the reduction of latency, leading to an enhanced and more seamless user experience. MIMO is a technology implemented in 5G communication systems to enhance network performance. This technology facilitates communication systems in transmitting multiple data streams concurrently via multiple antennas, resulting in enhanced data rates, improved coverage, and increased reliability^6^. Resource sharing in MIMO networks presents multiple challenges. The capacity of a MIMO network is specifically constrained by the number of deployable antennas, presenting challenges in resource sharing among multiple users^7^. Resource sharing in MIMO networks may result in contention issues stemming from elevated demand for bandwidth and data rates, which can lead to decreased throughput and heightened latency. MIMO systems must implement effective resource-sharing techniques^8^. This document outlines techniques including dynamic channel allocation, enabling efficient resource sharing among users, and load balancing, which guarantees equitable resource utilisation. Furthermore, network coding has the capability to decrease the volume of data required for transmission, leading to a reduction in overall resource consumption^9^.

MIMO systems are integral to 5G communication systems, offering enhanced performance characterised by improved data throughput, spectral efficiency, and heightened reliability. Resource sharing represents a significant characteristic of MIMO systems within 5G communication frameworks. This method facilitates the distribution of accessible spectral resources among users, optimising their utilisation. Resource sharing necessitates the allocation of resources to multiple users located in different geographic cells, each accessing a variety of services^10^. Resource allocation can effectively reduce interference and enhance network capacity. The process involves meticulous management of transmission power and throughput limitations for each subscriber, ensuring access to desired services with minimal disruptions. Resource sharing enables 5G communication systems to enhance flexibility in user access and service support^11^. Different users can maintain independent data flows while utilising the same resources, enabling simultaneous access to various services over the same network.

The services can be modified without obstructing the resources. Resource sharing facilitates the capability of 5G communication systems to accommodate various frequency bands and broader bandwidths^12^. This technology is applicable for advanced services, including autonomous vehicles and homes connected through the Internet of Things. Increased bandwidths facilitate enhanced data transmission capabilities, resulting in improved data speeds and a more dependable connection. Resource sharing is critical in 5G communication systems, as it enhances spectral efficiency and throughput, offers increased flexibility in service support, and facilitates the utilisation of wider bandwidths and multiple frequency bands^13^. The extensive utilisation of resource sharing is essential for the effective implementation of 5G communication systems. The advancement of 5G communication systems is transforming the methods by which resources are utilised and shared. MIMO technology represents a significant advancement in the development of 5G networks. This technology involves an antenna array configuration that facilitates the transmission and reception of data between devices using multiple antennas. The implementation facilitates enhanced spectral efficiency, elevated data rates, and superior system performance and reliability^14^. It facilitates enhanced resource sharing within 5G networks. A single radio frequency is capable of concurrently transmitting multiple data streams to various users. The system facilitates enhanced efficiency in the utilisation of the available spectrum, resulting in an increased data transmission rate. The implementation of multiple antennas enhances the signal-to-noise ratio, resulting in an improved quality of service (QoS) for users. MIMO technology enhances the scalability of 5G networks. The system enables the transmission of data through multiple antennas, thereby accommodating a greater density of users. The implementation can enhance coverage and performance in environments with high user densities^15^. This can reduce latency and enhance the user experience. MIMO technology in 5G networks represents a significant advancement that enhances the efficiency of resource sharing and utilisation within these networks. This technology enhances spectral efficiency, data rates, quality of service (QoS), and scalability, positioning it as a critical component for 5G networks. This research presents the following key contributions.

- The proposed model facilitates a more efficient utilisation of the available spectrum, resulting in enhanced data rates and improved overall performance.
- The proposed model has the capability to enhance the user capacity within a specified area. The outcome may result in enhanced system capacity and better coverage.
- The proposed model facilitates enhanced interference management, leading to improved signal-to-interference ratios and overall performance optimisation.
- The proposed model enhances coverage efficiency by facilitating the coverage of extensive geographical regions.
- The proposed model is designed to minimise latency by decreasing the necessity for handoffs among various cells. It has the potential to enhance user experience and optimise system performance.

The subsequent sections of the paper are structured as follows. Chapter II presents a comprehensive overview of the previous research studies. Chapter III presents the proposed model, while Chapter IV conducts a comparison between the existing and proposed models. Chapter V presents the results, while Chapter VI outlines the conclusion and future scope of the proposed framework.

## Related works

MIMO represents a vital technology within 5G communication systems, enhancing spectral efficiency, data rate, and coverage significantly. The system employs multiple antennas at both the transmitter and receiver to facilitate the simultaneous transmission and reception of multiple signals. The method enhances spectral efficiency by enabling the transmission, reception, and decoding of multiple signals at a single frequency. Hu, R. Q., et al.^16^ have presented a system that employs advanced radio technologies and protocols to enhance spectrum efficiency and energy conservation in cellular networks. The system facilitates a cohesive environment for various radio technologies enabling them to coexist and allocate resources in a synchronised fashion. The optimisation of the total spectrum and energy savings is ensured. The framework facilitates a more dynamic and adaptive network, optimising resource utilisation while reducing energy consumption. The system facilitates efficient spectrum and energy utilisation through the implementation of multiple access points, such as small cells, multi-antenna systems, and various advanced radio technologies. Luoto, P., et al.^17^ discusses co-primary multi-operator resource sharing for small cell networks as a form of cooperative communication where multiple cellular service providers utilise a common set of radio resources. This resource-sharing method enables multiple operators to utilise the same frequency spectrum while delivering their services independently. Sharing resources enables operators to decrease capital and operational expenses, thereby enhancing coverage and capacity for their customers. Georgakopoulos, A., et al.^18^ discuss that resource sharing in 5G contexts refers to the capability of multiple users to access identical resources over a shared network. Ensuring the network operates efficiently and cost-effectively is essential. Resource sharing minimises the energy and resource requirements for network operation, thereby lowering the overall operational costs associated with the network. Kliks, A., et al.^19^ have discussed that resource sharing in 5G networks presents a significant challenge for operators and service providers. 5G networks exhibit high throughput, low latency, and enhanced reliability, necessitating substantial resources including spectrum, bandwidth, and computing power. To enhance operational efficiency, it is essential for operators and service providers to facilitate the secure and efficient sharing of resources. Lloret, J., et al.^20^ discuss an approach utilising multi-access edge computing (MEC), which positions computing resources in proximity to the user, thereby reducing latency and enhancing performance. Additionally, 5G networks leverage virtualisation and cloud computing to facilitate resource sharing among various operators and service providers. Network operators can utilise artificial intelligence and machine learning to enhance resource utilisation and performance efficiency.

Thakre, P. N., et al.^21^ have analysed the allocation of transmit power to each user to optimise system throughput and ensure fairness among users. The process involves the implementation of a power allocation algorithm, which distributes the total available power among users. This distribution aims to maximise overall throughput while adhering to fairness constraints. The algorithm incrementally allocates additional power to the user experiencing the lowest power levels and continues this process until the total transmit power capacity is fully utilised. This mechanism guarantees equitable distribution of power among all users while optimising overall system throughput. Naeem, M., et al.^22^ discuss the utilisation of multiple antennas for signal transmission and reception, resulting in enhanced throughput and minimised interference. Reinforcement learning has been applied to enhance the performance of MIMO systems, enabling the system to adjust to varying environmental conditions and user requirements. Qian, M., et al.^23^ discusses a centralised network architecture for 5G mobile communication systems that employs super base stations, representing an innovative approach to wireless communication. The super base stations are engineered to deliver improved coverage and capacity through a centralised network architecture. The super base station is designed as a high-power base station capable of managing multiple frequency bands, thereby facilitating more efficient and reliable connections. Logeshwaran, J., et al.^24^ discuss that resource sharing can enhance network performance by enabling multiple users to access the same resources concurrently, rather than requiring separate resources for each user. Resource sharing facilitates a more efficient utilisation of the network’s resources, leading to a reduction in outages, enhancement of performance, and a decrease in costs. The reduction in the network’s environmental impact is achieved through the decreased consumption of resources required for network power. Kiruthiga, T., et al.^25^ discuss the reduction of data transmitted over the network, leading to decreased energy consumption of the network. In-network data aggregation serves to minimise the volume of data transmitted to the base station, decrease network traffic, and lower the energy consumption of the nodes. In-network data aggregation facilitates the consolidation of data from various nodes into a singular packet or the distribution of data from an individual node into multiple packets. The system is capable of compressing data, filtering out extraneous information, and executing statistical operations on the dataset.

Yarkina, N., et al.^26^ discuss that the cellular system is engineered to enable multiple tenants to utilise the same radio access network resources, while guaranteeing that each tenant achieves the performance level that has been assured to them. This system operates on the principle of slicing, which involves partitioning the entire network into several virtual networks. Each virtual network is allocated its own resources and configured with specific policy settings. The system employs a priority-based methodology for resource allocation to each tenant. The system enables tenants to achieve varying degrees of performance isolation based on their assigned priority level. Lloret, J., et al.^27^ have examined underwater wireless sensor communications operating within the 2.4 GHz ISM frequency band, adhering to the IEEE 802.15.4 standard. The frequency band exhibits minimal interference, facilitating effective sound signal propagation in underwater environments. Communications via underwater wireless sensors operating in the 2.4 GHz ISM frequency band present multiple benefits. The communication system, characterised by low power consumption and low data rates, is appropriate for a range of applications. Benzaghta, M., et al.^28^ have presented a system that employs a substantial number of antennas to enhance the performance of the network. The system employs multiple antennas at both the base station and the user device to transmit and receive multiple signals simultaneously. This configuration facilitates the transmission of increased data volumes and extends the range of wireless signals. The objective is to deliver increased data rates, enhanced reliability, and reduced latency for users of 5G technology. Table 1 shows the comprehensive analysis. From the above comprehensive analysis, the following issues were identified. They are the,

- In MIMO systems, the signals transmitted by the multiple antennas interfere, reducing the overall system performance.
- Estimating the channel in MIMO systems is more complex than in traditional single-antenna systems.
- In MIMO systems, the signals transmitted by the multiple antennas must be synchronized to maximize the system’s performance.
- MIMO systems are more vulnerable to eavesdropping and other security threats due to the increased complexity of resource sharing.
- MIMO systems are more susceptible to noise due to the increased number of antennas.

The novelty of proposed research work has highlighted as the following,

- Adaptive beamforming technique that adjust based on real-time user load distribution. This improves spectral efficiency by optimizing antenna radiation patterns for high-load users while minimizing interference.
- Load based dynamic slicing for MIMO resources, prioritizing users based on QoS demands and network load. This ensures fair resource allocation among multiple tenants while maintaining isolation.
- Integration of Non-Orthogonal Multiple Access with MIMO to enhance user multiplexing under varying load conditions. This increases user capacity and reduces latency by allowing non-orthogonal resource sharing in overloaded cells.
- Model interference and load distribution across MIMO antennas, optimizing handovers and resource blocks. This reduces signaling overhead and enhances load balancing in ultra-dense 5G networks.
- Dynamic switching between single-user and multi-user based on traffic load and energy constraints. This Reduces power consumption in low-load scenarios while maintaining high throughput in congested networks.

**Table 1:** Comprehensive analysis.

Authors	Year	Model/architecture	Resource allocation	Resource allocation	Load management
Hu et al.^16^	2014	Wireless Heterogeneous Network (5G)	Energy and spectrum-efficient allocation	Optimized spectrum and energy use	Dynamic load balancing across heterogeneous networks
Luoto et al.^17^	2015	Co-primary multi-operator small cell network	Shared spectrum among operators	Improved spectrum efficiency	Cooperative load distribution among operators
Georgakopoulos et al.^18^	2016	Sustainable 5G resource-sharing model	Energy and resource-efficient allocation	Enhanced sustainability via sharing	Dynamic load adaptation for efficiency
Kliks et al.^19^	2018	5G network resource-sharing framework	Multi-operator resource sharing	Efficient spectrum and infrastructure use	Decentralized load management
Lloret et al.^20^	2011	WSN for vineyard monitoring	Image processing-based allocation	Energy-efficient sensor usage	Task-based load distribution
Thakre et al.^21^	2022	PD-NOMA for 5G	Power-domain allocation	Improved spectral efficiency	User clustering for load balancing
Naeem et al.^22^	2021	RL/DL-based MIMO system	AI-driven dynamic allocation	Optimized MIMO resource usage	Adaptive load control via AI
Qian et al.^23^	2015	Super Base Station (5G)	Centralized resource management	High-capacity utilization	Centralized load coordination
Logeshwaran et al.^24^	2019	D2D communication	Enhanced D2D resource allocation	Improved proximity-based reuse	Interference-aware load management
Kiruthiga et al.^25^	2021	WSN data aggregation	In-network aggregation	Energy-efficient data transmission	Reduced communication load
Yarkina et al.^26^	2022	Multi-tenant 5G slicing	Priority-based slice allocation	Equitable resource sharing	Performance-isolated slice management
Lloret et al.^27^	2012	Underwater WSN (2.4 GHz)	Bandwidth-constrained allocation	Low-frequency band utilization	Delay-tolerant load handling
Benzaghta et al.^28^	2021	Massive MIMO (5G)	Channel estimation-based allocation	High spatial efficiency	User-centric load distribution

Table 2 provides the novelty analysis of proposed research,

**Table 2:** Novelty analysis.

Novelty	Key technology	Performance gain
Dynamic load-aware beamforming	Adaptive precoding	Higher spectral efficiency, reduced interference
AI-driven multi-tenant slicing	Load based resource allocation	Fair QoS, isolation in multi-operator networks
Hybrid NOMA-MIMO sharing	Power-domain multiplexing	Increased user capacity, lower latency
Load balancing	Interference modeling	Efficient handovers, reduced overhead
Load-adaptive MIMO switching	SU/MU switching	Energy savings, adaptive throughput

The MIMO can be used to achieve spatial multiplexing, which allows multiple data streams to be transmitted over the same channel. It is achieved by using multiple antennas to encode and decode multiple streams, allowing for increased data rates. Also, the MIMO can be used to increase the range of the signal, as multiple antennas can be used to transmit the signal over multiple paths and beams, increasing the signal’s range.

## Proposed model

**Fig. 1:**
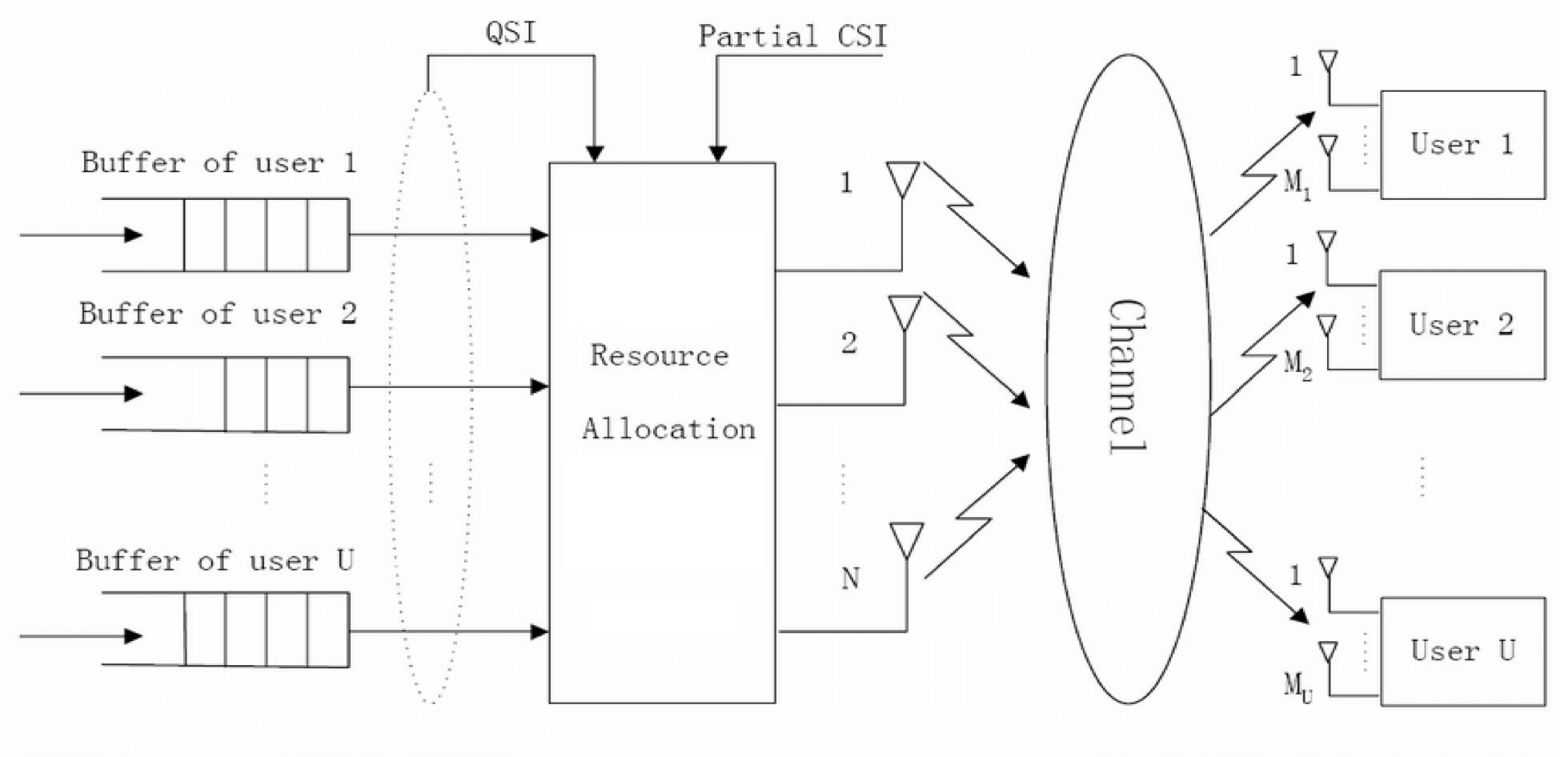
Resource allocation for MIMO users.

The 5G communication systems are expected to bring higher data rates and better reliability by utilizing MIMO systems. A resource-sharing framework is required to realize the full potential of MIMO systems. This resource-sharing framework ensures that different MIMO users can effectively and efficiently share available resources. It also ensures that the resources are used optimally and that the overall quality of service is maintained. The resource-sharing framework consists of two components: the resource-sharing protocol and the resource-sharing algorithm. The resource allocation has expressed in the following (Fig. 1).

The resource-sharing protocol defines the rules and conditions of resource sharing, while the resource-sharing algorithm allocates the resources. The resource-sharing algorithm considers the traffic load, channel conditions, and other factors to distribute the resources optimally. The resource-sharing framework also includes a mechanism for preventing interference between MIMO users. It is accomplished by applying various techniques. These techniques allow the MIMO users to communicate without interfering with each other.

### Proposed algorithm

Load-based resource sharing Algorithm (LBRS) developed for MIMO systems in 5G communication. A distributed and decentralized resource allocation algorithm allows nodes to share resources based on Load. The LBRS algorithm is designed to improve the overall performance of a MIMO system by reducing the interference between nodes and increasing the overall throughput. It allows nodes to adjust their transmit power and antenna selection based on their current Load. It is done by having nodes broadcast their current load information to other nodes in the system. Then, nodes can adjust their transmit power and antenna selection to reduce interference and improve system performance. The LBRS algorithm is designed to be low complexity and provide real-time feedback. It is also designed to be robust against malicious nodes, as the broadcasted load information can be used to detect malicious nodes.

The LBRS algorithm is readily implementable within current MIMO systems, facilitating a quicker and more efficient deployment process. The proposed algorithm aims to minimise interference, enhance overall system performance, and facilitate straightforward integration into current systems. Load-based resource-sharing algorithms are implemented in MIMO systems for 5G communication to enhance the efficiency of resource allocation among users. This algorithm optimises radio resource utilisation by allocating resources to users according to their current load conditions. The system allocates resources based on user load, providing increased resources to users with higher demands and reducing allocations for those with lower demands. The algorithm contributes to the reduction of interference and enhancement of network performance. The algorithm provides significant utility in resource scheduling, enabling the network to dynamically allocate resources to users in accordance with their current load and prevailing system conditions. The process guarantees an enhancement in user experience while optimising resource utilisation. Additionally, the algorithm is capable of enhancing the system’s spectral efficiency through the effective utilisation of resources. The algorithm.1 has expressed the functions of Load based resource-sharing algorithm.

**Algorithm 1:**
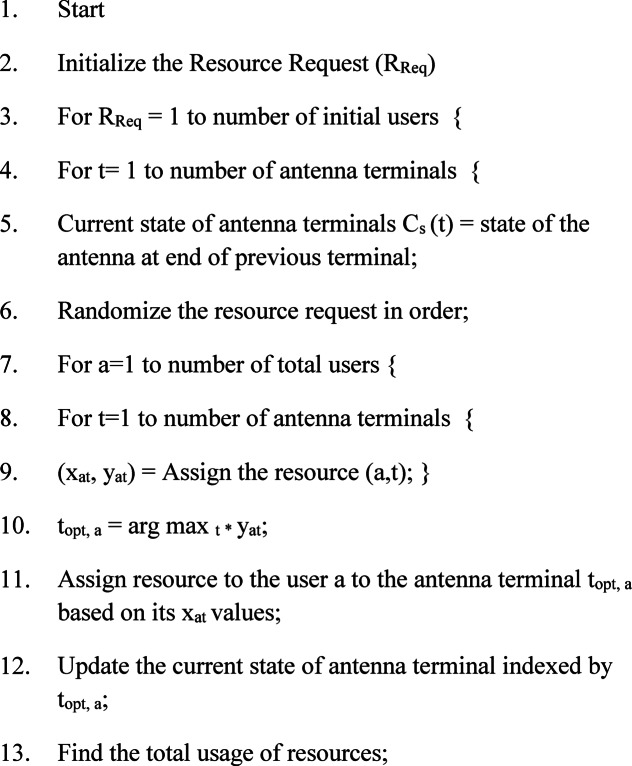
Load based resource sharing algorithm (LBRS).

Load-based resource-sharing algorithm uses distributed learning techniques to adjust users’ resource allocation in the system dynamically. It works in two stages – first, the system learns the characteristics of each user’s traffic; second, it allocates resources to users based on the traffic characteristics. In the learning stage, the system measures the average rate at which data is sent or received for each user and the average amount of packet loss. Based on this information, the system learns the best resource allocation for the users. In the resource allocation stage, the system assigns resources to each user to maximize the system’s overall performance. It is done by allocating resources taking into account the traffic characteristics of each user.

Let’s consider a MIMO system with the base station equipped operator and different antenna terminals. The transmitting antenna indicated as A_t_ and receiving antenna indicated as A_r_. Here the A_t_ > 1 with all the independent data streams (D_s_ < A_t_) for all the resource allocation stages. Now the active resource request (R_req_A_) he coverage region is (|R_req_A_| > A_t_). The discrete time form of the MIMO request has mentioned the following Eq. 1

1 $${A_m}=(E_{m}^{s}*T)+{d_m}$$

Where, E^s^_m_ represents the channel vector of transmission antenna terminal, m is the active user request. Now the estimation of transmit symbol T can be expressed as the following,

2 $$T=\sum\limits_{{m=1}}^{n} {{x_m}*\left\{ {\sqrt {{f_m}} *{P_m}} \right\}}$$

The noise from the receiver terminal has indicated in d_m_. This has shown in the Eq. 3

3 $${d_m}=\sum\limits_{{n \ne m}} {(E_{m}^{s}*{x_n})*(\sqrt {{f_n}} *{P_n})+{d_n}}$$

Sub.Eqs. 2 and 3 in Eq. 1

4 $${A_m}=(E_{m}^{s}*\sum\limits_{{m=1}}^{n} {{x_m}*\left\{ {\sqrt {{f_m}} *{P_m}} \right\}} )+(\sum\limits_{{n \ne m}} {(E_{m}^{s}*{x_n})*(\sqrt {{f_n}} *{P_n})+{d_n}} )$$

Now the zero padding precoding terminal vectors for the resource requests may fulfill the following requirements.

5 $$\{ E_{m}^{s}*{T_m}\} ={\mu _m};{\mu _m} \ne 0,m \in {R_{req}}$$

6 $$\{ E_{m}^{s}*{T_n}\} =0;\forall n \in {R_{req}}$$

Resource allocation is determined by the user’s average rate and the incidence of packet loss. The system allocates resources based on user rates, ensuring that those with higher rates are provided with more resources, while those with lower rates receive fewer resources. The process operates in two distinct stages: initially, the system analyses user traffic characteristics; subsequently, it allocates resources to users in accordance with these traffic characteristics. The system allocates resources based on user rates, ensuring that those with higher rates receive a greater share, while those with lower rates receive less. This allocation strategy enhances the overall performance of the system.

## Comparative analysis

The proposed Load based resource sharing framework (LBRS) has compared with the existing Cost-Efficient Resource Sharing (CERS), intelligent resource sharing scheme (IRSS), balanced partitioning dynamic cluster head algorithm (BPDCA) and Optimal Cluster Head Selection algorithm (OCHSA). Here, the network simulator (NS-2) is the simulation tool used to execute the results and load balancing dataset^29^ is used. The following Table 3 expresses the simulation setup details.

**Table 3:** Simulation setup.

Parameter	Value
Surface of the simulation	2500 m*1800m
Inter-frame transmission duration	50 s
DRT (data rate – transmission)	8 Mbps
SIR (signal interference rate)	20 ms
TW_p_ (transmission window period)	22 ms
MIMO antenna bandwidth	800 MHz
Antenna carrier frequency	15.6 GHz
Simulation interval	15 s
No.of MIMO terminals	8

- Surface area (2500 m × 1800 m) represents a medium-sized urban or campus environment, typical for 5G MIMO system testing. It ensures realistic path loss, shadowing, and interference modelling.
- Inter-frame transmission duration (50 s) chosen to evaluate long-term network stability under sustained traffic. It helps analyze latency and queuing effects in multi-user MIMO scenarios.
- Data rate – transmission (DRT: 8 Mbps) represents a moderate QoS requirement. It balances realism and computational feasibility for large-scale simulations.
- Signal interference Rate (SIR: 20 ms) models interference mitigation intervals in dense deployments. It matches 5G NR scheduling timelines (1 ms–20 ms slots).
- Transmission window period (TWp: 22 ms) reflects TCP/IP-like congestion control in MIMO link adaptation. It ensures fairness in multi-user scheduling.
- Simulation Interval (15 s) balances transient vs. steady-state analysis. It captures channel coherence time variations in mobility scenarios.
- Number of MIMO Terminals (8) represents a small-to-medium cell. It aligns with MU-MIMO practical limits.

### Computation of Delta-P (Δp)

The load-based resource-sharing framework is a critical element of 5G communication systems. It enables efficient utilization of the limited resources available in a communication system. In order to optimize the use of these resources, the Δp (Delta-P) analysis of resource sharing for MIMO systems can be used. The Δp is a mathematical tool that enables the evaluation of the resource-sharing performance of a MIMO system in terms of the amount of power consumed by each of the transmission antennas. The Δp is based on a mathematical model of the MIMO system, which defines the relationship between the total power transmitted by the system and the power consumed by each of its antennas. The number of antennas and their respective powers determine the total power.

7 $$\Delta p=\left( {\frac{{{R_{req}}({P_t})}}{{{R_{req}}({P_t})+{R_{req}}({P_f})}}} \right)+\left( {\frac{{{R_{req}}({N_t})}}{{{R_{req}}({N_t})+{R_{req}}({N_f})}}} \right) - 1$$

Where the R_req_(P_t_) represents the resource request (positive true), R_req_(P_f_) represents the resource request (positive false), R_req_(N_t_) represents the resource request (negative true) and R_req_(N_f_) represents the resource request (negative false). The Table 4 shows the comparison of delta-p (in terms of terminal access) between the existing CERS, IRSS, BPDCA, OCHSA models and proposed LBRS.

**Table 4:** Comparison of Δp-T (in %).

No.of terminals	CERS (T)	IRSS (T)	BPDCA (T)	OCHSA (T)	LBRS (T)
T_1_	56.73	52.71	68.66	67.29	89.77
T_2_	57.78	53.72	69.80	68.21	89.34
T_3_	58.49	54.65	70.91	69.54	90.58
T_4_	59.43	55.63	72.04	70.60	90.71
T_5_	60.31	56.60	73.16	71.72	91.11
T_6_	61.19	57.57	74.29	72.85	91.52
T_7_	62.07	58.54	75.41	73.97	91.92
T_8_	62.95	59.51	76.54	75.10	92.33

The Table 5 shows the comparison of delta-p (in terms of Resource transmission) between the existing CERS, IRSS, BPDCA, OCHSA models and proposed LBRS.

**Table 5:** Comparison of Δp-R (in %).

No.of terminals	CERS (*R*)	IRSS (*R*)	BPDCA (*R*)	OCHSA (*R*)	LBRS (*R*)
T_1_	59.79	55.65	71.61	70.41	90.69
T_2_	60.67	56.62	72.74	71.54	91.10
T_3_	61.66	57.60	73.73	72.60	91.50
T_4_	62.65	58.57	74.73	73.67	91.90
T_5_	63.58	59.54	75.79	74.76	92.30
T_6_	64.54	60.52	76.82	75.85	92.71
T_7_	65.50	61.49	77.86	76.93	93.11
T_8_	66.45	62.46	78.89	78.01	93.51

**Fig. 2:**
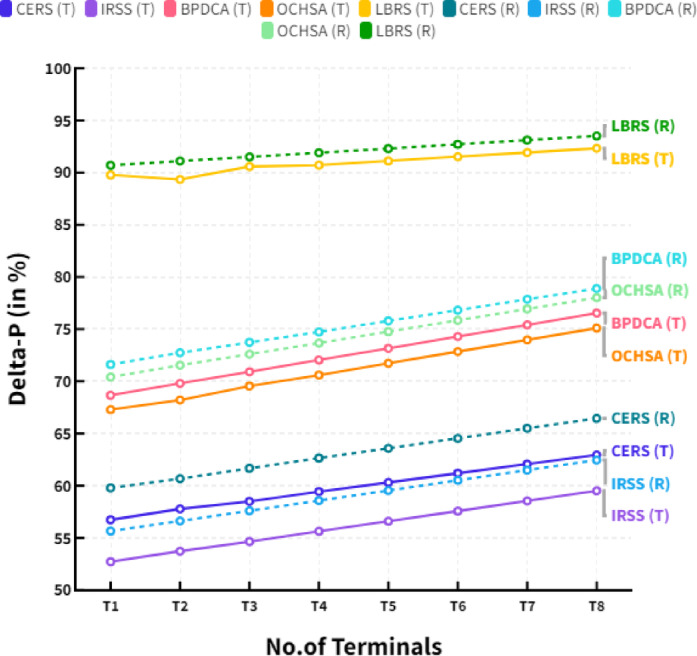
Comparison of delta-P in different terminals.

Figure 2 shows the comparison of Delta-P in different terminals. The straight lines in figure show the analysis in terms of terminal access and dotted lines expressed the resource transmission in MIMO systems. The Δp analysis enables the evaluation of the power consumed by each antenna by considering the changes in the power transmitted by the system. It helps to identify the optimal power settings for each antenna in the system, which can be used for efficient resource sharing. The Δp analysis can also identify MIMO systems’ most efficient resource management strategies. For example, it can be used to identify the optimal power settings for each antenna in order to maximize the overall throughput.

### Computation of prevalence threshold

The prevalence threshold of resource sharing for MIMO systems in 5G communication systems using load based resource sharing framework is determined by the system configuration and the user preference. Generally, resource sharing can be triggered when the load on the system is higher than a certain threshold or when the user requests a resource switch. This threshold depends on the system resources available, the number of users, the user preferences, and the system traffic. Generally, the resource sharing threshold should be set at a level that ensures that the system resources are efficiently shared among users, while also providing an acceptable level of performance.

8 $${P_{th}}=\left\{ {\frac{{\sqrt {\left( {\frac{{{R_{req}}({P_f})}}{{{R_{req}}({P_f})+{R_{req}}({N_t})}}} \right)} }}{{\sqrt {\left( {\frac{{{R_{req}}({P_f})}}{{{R_{req}}({P_f})+{R_{req}}({N_t})}}} \right)} +\sqrt {\left( {\frac{{{R_{req}}({P_t})}}{{{R_{req}}({P_t})+{R_{req}}({N_f})}}} \right)} }}} \right\}$$

Where the R_req_(P_t_) represents the resource request (positive true), R_req_(P_f_) represents the resource request (positive false), R_req_(N_t_) represents the resource request (negative true) and R_req_(N_f_) represents the resource request (negative false). The Table 6 shows the comparison of Prevalence threshold (in terms of terminal access) between the existing CERS, IRSS, BPDCA, OCHSA models and proposed LBRS.

**Table 6:** Comparison of prevalence threshold (in %).

No.of terminals	CERS (T)	IRSS (T)	BPDCA (T)	OCHSA (T)	LBRS (T)
T_1_	53.92	49.72	65.88	63.68	87.64
T_2_	54.25	51.22	66.47	65.55	88.68
T_3_	55.59	52.33	67.45	66.38	88.81
T_4_	56.26	53.70	68.17	67.90	89.55
T_5_	57.09	55.00	68.95	69.25	90.13
T_6_	57.93	56.31	69.74	70.60	90.72
T_7_	58.77	57.61	70.52	71.95	91.31
T_8_	59.60	58.92	71.31	73.30	91.89

The Table 7 shows the comparison of Prevalence threshold (in terms of resource transmission) between the existing CERS, IRSS, BPDCA, OCHSA models and proposed LBRS.

**Table 7:** Comparison of prevalence threshold (in %).

No.of terminals	CERS (*R*)	IRSS (*R*)	BPDCA (*R*)	OCHSA (*R*)	LBRS (*R*)
T_1_	57.09	55.01	68.96	69.25	90.13
T_2_	57.93	56.31	69.74	70.60	90.72
T_3_	58.76	57.62	70.53	71.95	91.30
T_4_	59.60	58.92	71.31	73.30	91.89
T_5_	60.43	60.23	72.10	74.65	92.47
T_6_	61.27	61.53	72.88	76.00	93.06
T_7_	62.10	62.84	73.67	77.35	93.64
T_8_	62.94	64.14	74.45	78.70	94.23

**Fig. 3:**
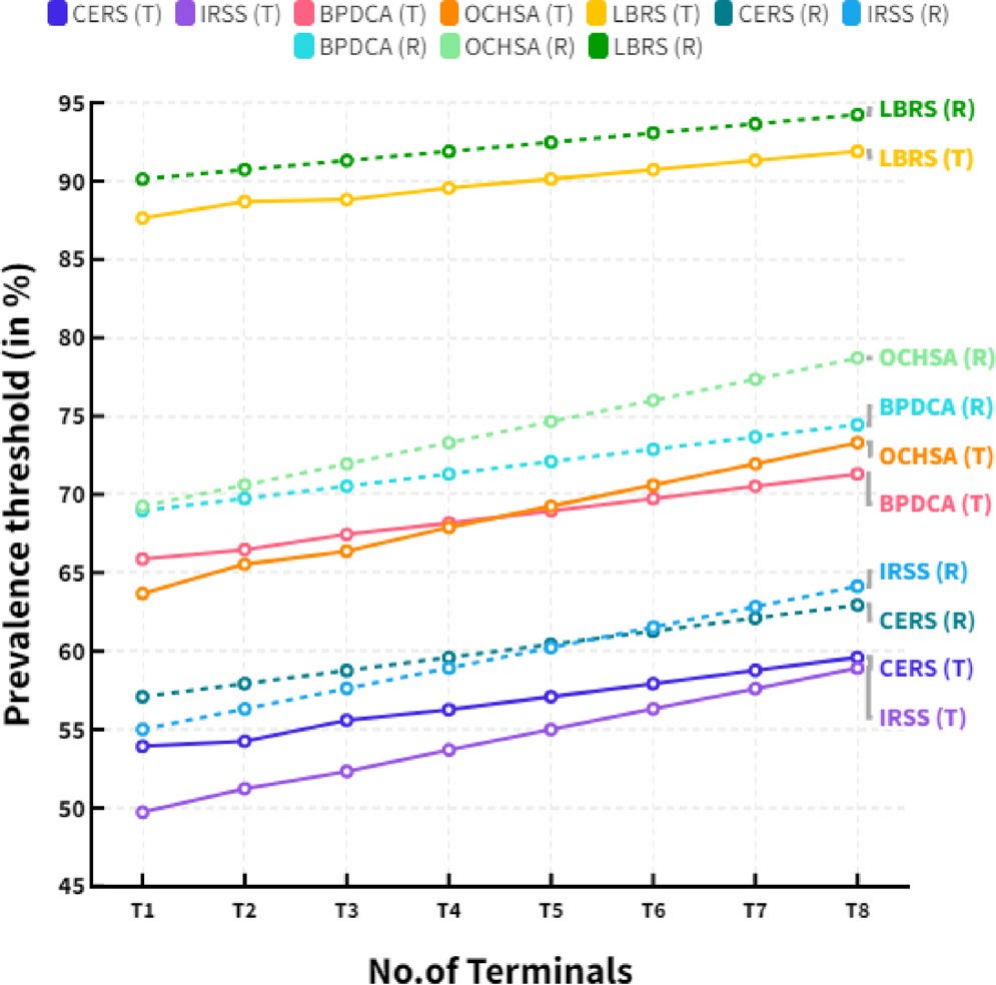
Comparison of Prevalence threshold in different terminals.

Figure 3 shows the comparison of Prevalence threshold in different terminals. The straight lines in figure show the analysis in terms of terminal access and dotted lines expressed the resource transmission in MIMO systems. The Prevalence Threshold of Resource Sharing (PTRS) for MIMO systems is a powerful tool for increasing the performance of MIMO systems. This technique allows the system to switch between multiple transmissions resources depending on channel conditions. By doing so, the system can exploit the best transmission resource available at any given moment, thereby improving the system’s performance. The PTRS technique helps reduce the error propagation rate since it enables the system to switch between different transmission resources based on the current channel conditions. It helps to reduce the impact of impairments, such as fading, multipath, and interference. In addition, using the PTRS technique, the system can better exploit the available resources and improve the system’s overall throughput. It, in turn, increases the system’s capacity and data rate.

### Computation of critical success index

The critical success index of resource sharing for MIMO systems in 5G communication systems using a load-based resource-sharing framework is highly dependent on the specific implementation of the system. Factors such as the number of users, network topology, channel characteristics, and the adopted resource-sharing algorithm can all contribute to the success of resource sharing.

9 $$CSI=\left( {\frac{{{R_{req}}({P_t})}}{{{R_{req}}({P_t})+{R_{req}}({N_f})+{R_{req}}({P_f})}}} \right)$$

Where the R_req_(P_t_) represents the resource request (positive true), R_req_(P_f_) represents the resource request (positive false), R_req_(N_t_) represents the resource request (negative true) and R_req_(N_f_) represents the resource request (negative false). The Table 8 shows the comparison of Critical success index (in terms of terminal access) between the existing CERS, IRSS, BPDCA, OCHSA models and proposed LBRS.

**Table 8:** Comparison of critical success index (in %).

No.of terminals	CERS (T)	IRSS (T)	BPDCA (T)	OCHSA (T)	LBRS (T)
T_1_	60.79	56.95	67.51	66.80	90.45
T_2_	62.09	57.95	68.21	67.88	90.61
T_3_	63.39	58.95	68.91	68.96	90.77
T_4_	64.69	59.95	69.61	70.04	90.93
T_5_	65.99	60.95	70.31	71.12	91.09
T_6_	67.29	61.95	71.01	72.20	91.25
T_7_	68.59	62.95	71.71	73.28	91.41
T_8_	69.89	63.95	72.41	74.36	91.57

The Table 9 shows the comparison of Critical success index (in terms of resource transmission) between the existing CERS, IRSS, BPDCA, OCHSA models and proposed LBRS.

**Table 9:** Comparison of critical success index (in %).

No.of terminals	CERS (*R*)	IRSS (*R*)	BPDCA (*R*)	OCHSA (*R*)	LBRS (*R*)
T_1_	65.99	60.95	70.31	71.12	91.09
T_2_	67.29	61.95	71.01	72.20	91.25
T_3_	68.59	62.95	71.71	73.28	91.41
T_4_	69.89	63.95	72.41	74.36	91.57
T_5_	71.19	64.95	73.11	75.44	91.73
T_6_	72.49	65.95	73.81	76.52	91.89
T_7_	73.79	66.95	74.51	77.60	92.05
T_8_	75.09	67.95	75.21	78.68	92.21

**Fig. 4:**
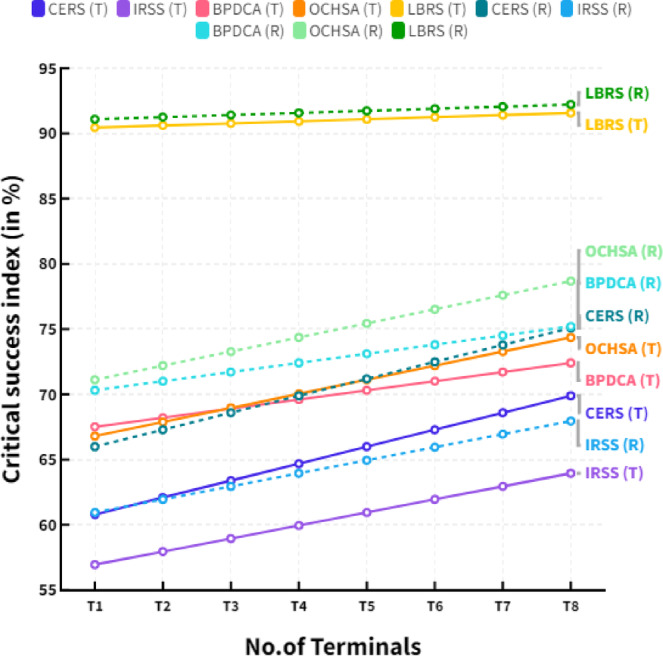
Comparison of Critical success index in different terminals.

Figure 4 shows the comparison of Critical success index in different terminals. The straight lines in figure show the analysis in terms of terminal access and dotted lines expressed the resource transmission in MIMO systems. Critical success index calculations of resource sharing for MIMO systems are essential because they provide a quantitative measure of the success of the resource-sharing process in achieving the desired objectives. It helps identify improvement areas and ensure that the sharing process is optimally configured. Furthermore, these calculations can be used to gauge the overall performance of the MIMO system, allowing greater insight into how the system is operating and how it can be improved.

### Computation of Matthews correlation coefficient

The Matthews correlation coefficient (MCC) is essential for evaluating resource-sharing algorithms for multiple-input multiple-output (MIMO) systems. MCC takes into account both the accurate positives (P_a_) and accurate negatives (N_a_) as well as forged positives (P_f_) and forged negatives (N_f_) when calculating the correlation between two variables. It allows for a better comparison between the two variables than other methods, such as accuracy or precision. MCC is a measure of the quality of a binary classifier, and it can be used to measure the quality of resource sharing for MIMO systems. The MCC is calculated as follows:

10 $$MCC=\left( {\frac{{\{ {R_{req}}({P_t})*{R_{req}}({N_t})\} - {R_{req}}({P_f})*{R_{req}}({N_f})}}{{\sqrt {\{ {R_{req}}({P_t})+{R_{req}}({P_f})\} *\{ {R_{req}}({P_t})+{R_{req}}({N_f})\} *\{ {R_{req}}({N_t})+{R_{req}}({P_f})\} *\{ {R_{req}}({N_t})+{R_{req}}({N_f})\} } }}} \right)$$

Where the R_req_(P_t_) represents the resource request (positive true), R_req_(P_f_) represents the resource request (positive false), R_req_(N_t_) represents the resource request (negative true) and R_req_(N_f_) represents the resource request (negative false). The Table 10 shows the comparison of Matthews correlation coefficient (in terms of terminal access) between the existing CERS, IRSS, BPDCA, OCHSA models and proposed LBRS.

**Table 10:** Comparison of Matthews correlation coefficient (in %).

No.of terminals	CERS (T)	IRSS (T)	BPDCA (T)	OCHSA (T)	LBRS (T)
T_1_	75.49	63.48	75.55	64.98	96.98
T_2_	74.00	61.51	73.13	62.78	94.99
T_3_	73.20	60.38	72.72	61.98	93.79
T_4_	71.94	58.69	70.97	60.25	92.06
T_5_	70.79	57.14	69.55	58.75	90.47
T_6_	69.65	55.59	68.14	57.25	88.87
T_7_	68.50	54.04	66.72	55.75	87.28
T_8_	67.36	52.49	65.31	54.25	85.68

The Table 11 shows the comparison of Matthews’s correlation coefficient (in terms of resource transmission) between the existing CERS, IRSS, BPDCA, OCHSA models and proposed LBRS.

**Table 11:** Comparison of Matthews correlation coefficient (in %).

No.of terminals	CERS (*R*)	IRSS (*R*)	BPDCA (*R*)	OCHSA (*R*)	LBRS (*R*)
T_1_	70.90	59.25	71.12	61.31	93.31
T_2_	69.86	58.80	68.80	59.88	91.88
T_3_	68.82	58.35	66.48	58.45	90.45
T_4_	67.78	57.90	64.16	57.02	89.02
T_5_	66.74	57.45	61.84	55.59	87.59
T_6_	65.70	57.00	59.52	54.16	86.16
T_7_	64.66	56.55	57.20	52.73	84.73
T_8_	63.62	56.10	54.88	51.30	83.30

**Fig. 5:**
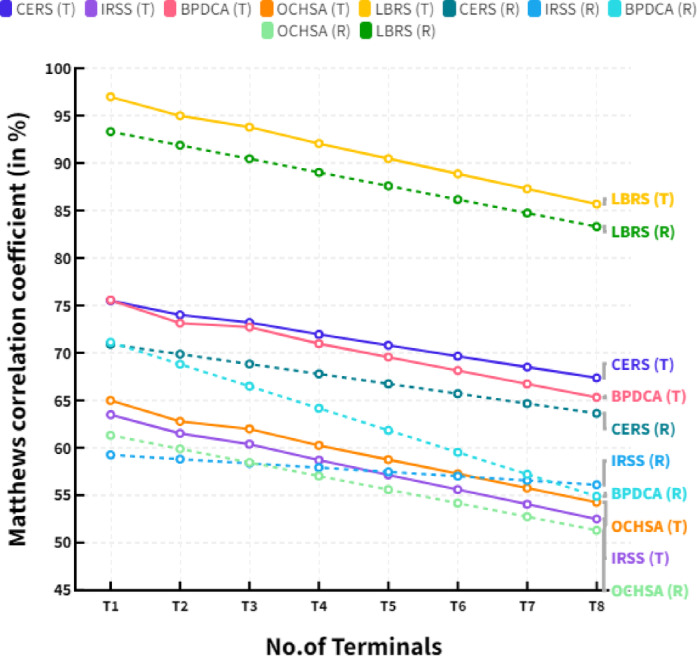
Comparison of Matthews’s correlation coefficient in different terminals.

Figure 5 shows the comparison of Matthews’s correlation coefficient in different terminals. The straight lines in figure show the analysis in terms of terminal access and dotted lines expressed the resource transmission in MIMO systems. The MCC also provides a better insight into the effectiveness of resource-sharing algorithms since it can help to identify areas of improvement in terms of false positives and false negatives. By identifying these areas, resource-sharing algorithms can be improved and optimized to serve MIMO systems’ needs better.

## Results and discussion

When a MIMO system is in a high interference environment, it can switch to a higher modulation scheme and coding rate to maximize data throughput. Similarly, when the system is in a low-interference environment, it can switch to a lower data rate to conserve energy. Now, the convergence of the proposed system performance has shown in the following Eq. 11

11 $$C=\left\{ {\frac{{\sum\limits_{{a=1}}^{b} {{R_a}(x)} }}{{\sum\limits_{{a=1}}^{b} {{T_a}(x)} }}} \right\}$$

Where, C represents the convergence of performance, ‘a’is indicated the initial resource request, ‘b’ is the end user resource request. ‘R’ represents the average value of the resource transmission and ‘T’ represents the total number of terminals in MIMO system. The convergence of overall performance has shown in the following (Table 12).

**Table 12:** Convergence of performance (in %).

Parameters	CERS (T)	CERS (*R*)	IRSS (T)	IRSS (*R*)	BPDCA (T)	BPDCA (*R*)	OCHSA (T)	OCHSA (*R*)	LBRS (T)	LBRS (*R*)
Δp	59.87	63.11	56.12	59.06	72.60	75.27	71.16	74.22	90.91	92.10
P_th_	56.68	60.02	54.35	59.58	68.56	71.71	68.58	73.98	89.84	92.18
CSI	65.34	70.54	60.45	64.45	69.96	72.76	70.58	74.90	91.01	91.65
MCC	71.37	67.26	57.92	57.68	70.26	63.00	59.50	56.31	91.27	88.31

**Fig. 6:**
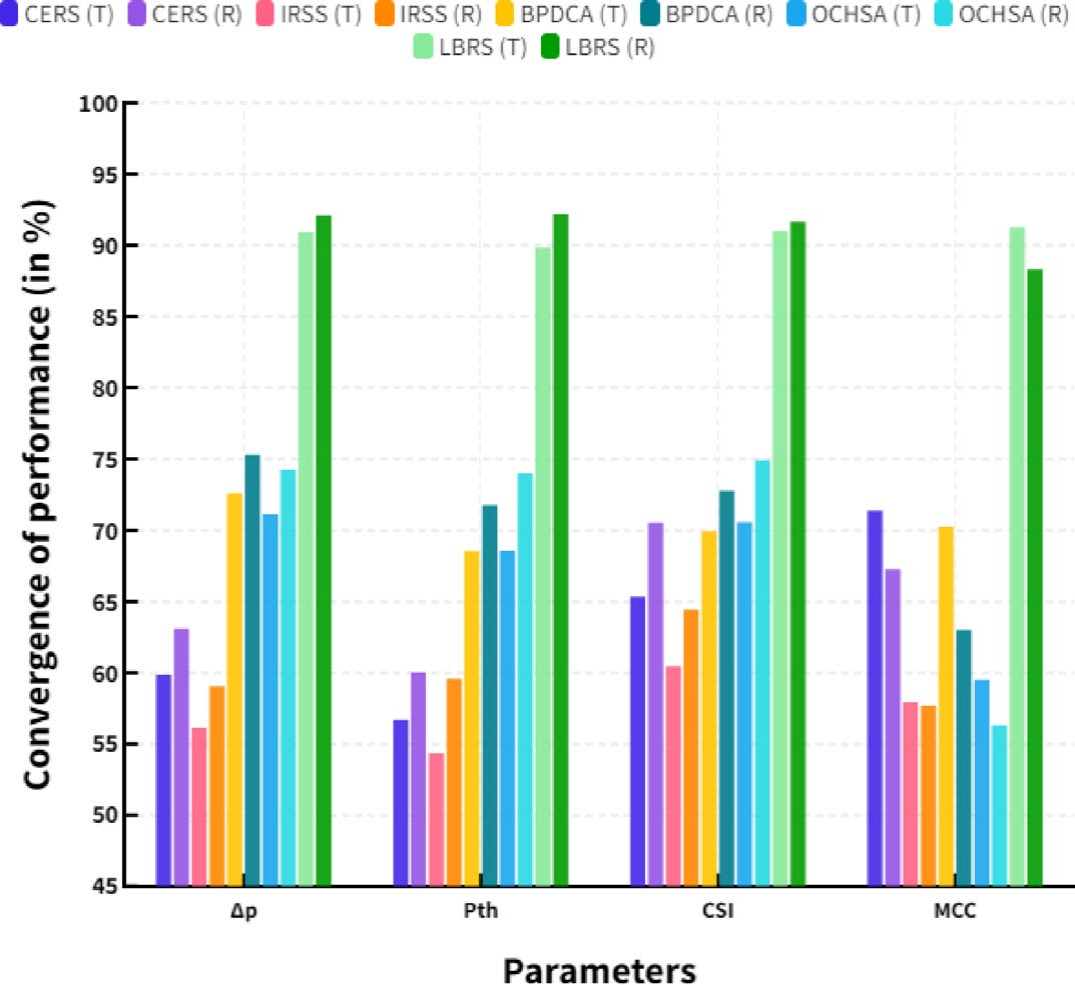
Convergence of performance.

Figure 6 shows the convergence of performance. In a comparison point, the proposed LBRS reached a 90.91% delta-P value, 89.84% prevalence threshold value, 91.01% critical success index value, and 91.27% Mathew’s correlation coefficient value at the terminal access. Meanwhile, the existing CERS obtained a 59.87% delta-P value, 56.68% prevalence threshold value, 65.34% critical success index value, and 71.37% Mathew’s correlation coefficient value, IRSS reached 56.12% delta-P value, 54.35% prevalence threshold value, 60.45% critical success index value and 57.92% Mathew’s correlation coefficient value, BPDCA achieved 72.60% delta-P value, 68.56% prevalence threshold value, 69.96% critical success index value and 70.26% Mathew’s correlation coefficient value and OCHSA reached 71.16% delta-P value, delta-P value, 68.58% prevalence threshold value, 70.58% critical success index value and 59.50% Mathew’s correlation coefficient value at the terminal access. In the same range, the proposed LBRS reached 92.10% delta-P value, 92.18% prevalence threshold value, 91.65% critical success index value, and 88.31% Mathew’s correlation coefficient value at the resource transmission. Meanwhile, the existing CERS obtained a 63.11% delta-P value, 60.02% prevalence threshold value, 70.54% critical success index value, and 67.26% Mathew’s correlation coefficient value, IRSS reached 59.06% delta-P value, 59.58% prevalence threshold value, 64.45% critical success index value and 57.68% Mathew’s correlation coefficient value, BPDCA achieved 75.27% delta-P value, 71.71% prevalence threshold value, 72.76% critical success index value and 63.00% Mathew’s correlation coefficient value and OCHSA reached 74.22% delta-P value, delta-P value, 73.98% prevalence threshold value, 74.90% critical success index value and 56.31% Mathew’s correlation coefficient value at the resource transmission. Hence mean value of the performance parameters is shown in the following Eq. 12

12 $$M={R_a} - {T_a}$$

Where, M represents the mean value, ‘R’ represents the average value of the resource transmission and ‘T’ represents the total number of terminals in MIMO system. The mean of overall performance has shown in the following (Table 13).

**Table 13:** Mean of performance (in %).

Parameters	CERS	IRSS	BPDCA	OCHSA	LBRS
Δp	3.24	2.94	2.67	3.06	1.19
P_th_	3.34	5.23	3.15	5.4	2.34
CSI	5.2	4	2.8	4.32	0.64
MCC	−4.11	−0.24	−7.26	−3.19	−2.96

**Fig. 7:**
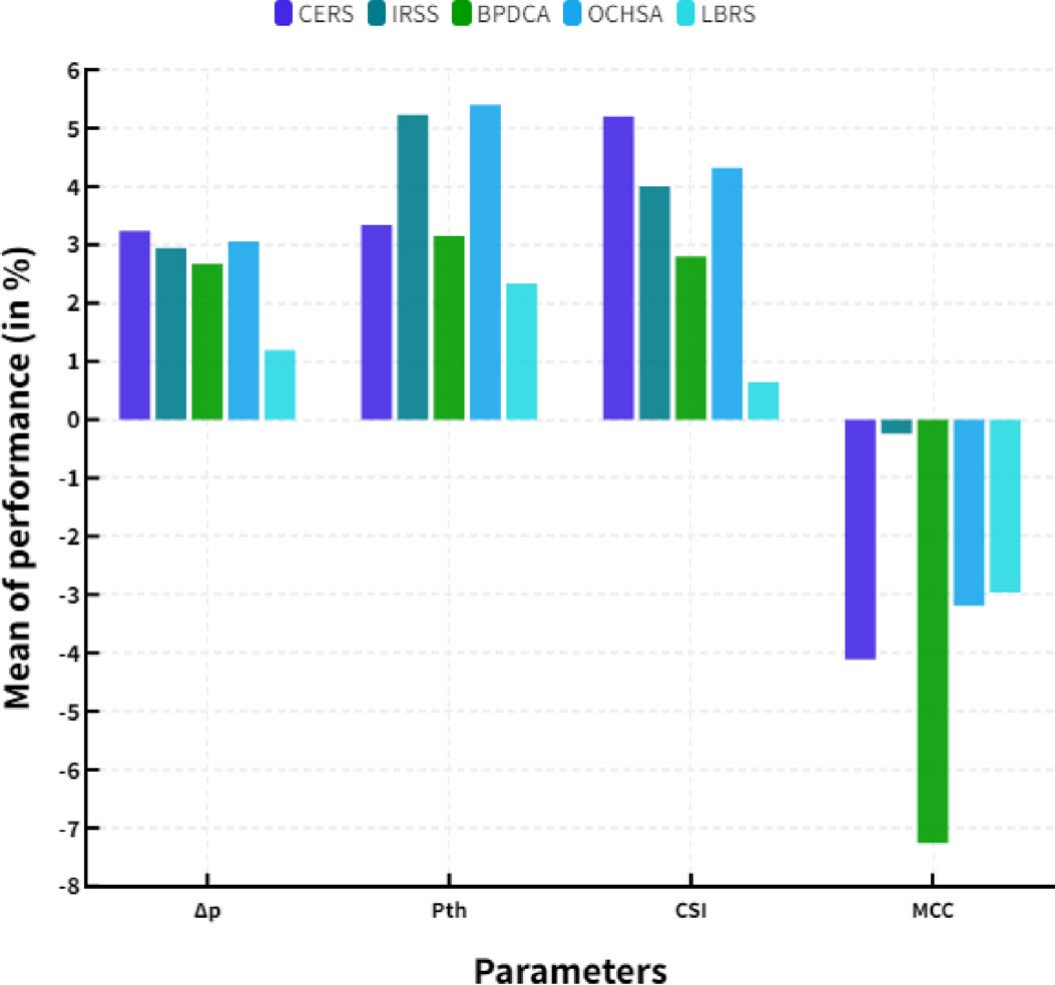
Mean of performance.

Figure 7 shows the mean of performance. MIMO systems can leverage beamforming techniques, in addition to the advantages provided by resource sharing. Beamforming facilitates the directional transmission of signals to optimise the signal-to-noise ratio. The system’s performance can be optimised through the utilisation of resource sharing. Resource sharing serves as an effective method for enhancing the performance of MIMO systems. The system is designed to adjust to varying environmental conditions, thereby optimising performance outcomes. Additionally, beamforming can be utilised alongside resource sharing to enhance the overall performance of the system.

## Conclusion

Load-based resource sharing for MIMO in 5G communication systems is the technique that efficiently utilizes the available spectrum and resources, allowing for higher data rates and better performance. It also helps prevent interference and ensures fairness among the users. The load-based sharing algorithm is based on fairness and efficiently balances the system’s load. The proposed LBRS reached a 90.91% delta-P value, 89.84% prevalence threshold value, 91.01% critical success index value and 91.27% Mathew’s correlation coefficient value at the terminal access, and 92.10% delta-P value, 92.18% prevalence threshold value, 91.65% critical success index value and 88.31% Mathew’s correlation coefficient value at the resource transmission. The performance of load-based resource sharing for MIMO in 5G communication systems is excellent. It has been tested in various scenarios and has been shown to perform better than other resource allocation techniques, such as equal sharing or maximum throughput. Tests have shown that it provides higher data rates and better spectral efficiency while maintaining user fairness. It can be used to increase the capacity of the system. The future scope of load-based resource sharing for MIMO in 5G communication systems is to provide more efficient and reliable usage of the available resources. By leveraging load-based resource sharing, 5G communication systems can optimize the network resources to increase throughput and reduce latency. Resource sharing can also improve the quality of service by allowing users to access higher data rates when needed. It can also help to reduce interference between different users, thereby improving the overall network performance.

This study also aligns with the United Nations Sustainable Development Goals (SDGs), particularly SDG 9 (Industry, Innovation, and Infrastructure), SDG 11 (Sustainable Cities and Communities), and SDG 12 (Responsible Consumption and Production). The proposed load-based resource-sharing framework enhances the performance and efficiency of 5G MIMO systems, contributing to the development of smarter, more sustainable, and reliable communication infrastructures essential for future smart cities and digital services.

## Data Availability

The data used to support the findings of this study are included in the article.
